# On a New Preparation of Pepsine

**Published:** 1870-03

**Authors:** Edward Long

**Affiliations:** Dublin


### ON A NEW PREPARATION OF PEPSINE.

By EDWARD LONG, L.A.H., Dublin.

During some recent investigations into the manufacture, relations, and general properties of pepsine, I was induced to make a solution in glycerine, somewhat after the manner of "Ellis' Rennet Wine," so much prescribed of late in this city. Many considerations led up to this idea, and the results more than realized my expectations.

In the ordinary process for making pepsine, there is necessarily used a considerable quantity of starch to produce a dry powder, which evidently opens the door to great adulteration, as it is difficult to detect the relative proportion of the active and passive constituents. As a consequence, we hear most discrepant opinions of the therapeutical value of pepsine. Some maintain that it is inert, as it often is, no doubt, through fraud or bad manipulation. Others, again, attach the importance to it which might be expected from reasoning on well-known physiological facts, and find their *a priori* conclusions supported by experience. The difference of opinion is due, in this case, to the difference in the quantity of pepsine present in an active state in the preparations used; and this must arise from some defect in the *modus operandi* of procuring it.

I will not here enter into any critique on the ordinary processes. A fair sample of pepsine may, with care, be obtained by most of those published, though erroneous statements have obtained currency as to the effect of various re-agents on it. What is desirable is that some convenient and certain process be discovered, with definite results easily tested.

With these objects in view, I commenced my experiments, and with the result of procuring a solution of pepsine definite and certain in strength, and so pleasant to the taste that even children will not find it objectionable, but the contrary.

The fresh stomach of the pig, having been washed in water sufficiently to remove all particles of food, and the cardiac end removed, as yielding rennet only, is cut into slips and digested for one week in as much glycerine as will entirely cover it, then strained and filtered. The resulting fluid is about the consistency of simple syrup, somewhat thinner than glycerine, of a pale, sherry color, sweet to the taste, with the characteristic flavor of pepsine.

A drachm of this, with 15 minims of muratic acid, and an ounce of water added, at 100°, readily dissolved 700 grains of moist fibrine, properly prepared; while the best of the solutions in general use, which I have tried, only dissolved 75 grains, and that not so completely under the same conditions.

Lactic acid, which is generally stated to answer as well as muriatic, I have found, comparatively inert, which tends to corroborate the original opinion of Prout in opposition to the statements of M. Bernard and Barreswill.

The solution I have described has this further recommendation, that whereas the various solutions of pepsine in sherry, etc., are very liable to spoil, this keeps very well. I have before me now some of it made seven months ago, in which there is no sign of decomposition.

Half a drachm to a drachm, taken in a little water, at or immediately after meals, ought to be found the most effective preparation of pepsine in use. It may be taken undiluted if preferred, but should always be associated with food in the stomach. It is quite compatible with most articles of food in ordinary use which are really wholesome and digestible; but it would be well to bear in mind that alcohol, unless very dilute, destroys pepsine, whether it be the natural exudation from the gastic mucous membrane, or that introduced as a suppliment by modern science. The cases in which it will be found applicable I leave to the physician, as it would be foreign to the purport of this paper to describe them.

As the action of any agent derived from the animal kingdom is an unknown quantity in calculating effects, its adoption, therapeutically, should be with discretion. I have, therefore, thought it right, considering the powerful solvent or digestive action of this solution, to test it in my own person before recommending it to others; and I can testify, for the confidence of my medical friends, that it is not only quite safe, but, so far as one can estimate in a limited experience, beneficial in the degree to be expected *a priori*.

To prevent confusion, and, at the same time, to invite trial, I have called my solution "Prepared Gastric Juice," or "Digestive Solution of Pepsine," a small bottle of which I shall be happy to send to any medical man disposed to try it—not with a view to elict clap-trap testimonials on narrow premises, but to enable them to judge of the value of this remedy in some of the most troublesome cases which come under the care of the physician.

Let it once be granted that pepsine, removed from under the action of the vital force, retains its activity, and it follows that simplicity and certainty in the process for obtaining it in such a form as will keep reasonably well are all that is required. Both desiderata I believe I have arrived at, judging by the tests already mentioned, and I now with confidence offer the result to the profession.

As a postscript it may not be unnecessary to remind that pepsine and rennet are two different things, and produce different effects; one of the most obvious is that the latter coagulates albumen, while the former dissolves the curd so formed. It has frequently come to my knowledge that people have been directed by their medical attendants to make whey with pepsine wine, and are not a little disappointed to find they can not succeed ; rennet wine or essence no doubt being intended.—*Dublin Medical Press and Circular.*—PHARMACIST.

